# Hybrid pin penetration open stent technique: Retrospective study of a new approach to frozen elephant trunk procedure

**DOI:** 10.1016/j.jvscit.2026.102505

**Published:** 2026-09-07

**Authors:** Chih-Chen Kao, Yao-Kuang Huang, Imin Tseng, Chien-Chao Lin, Ho-Cheng Kuo, Tsung-Shun Wang, Chu-Hsueh Lu

**Affiliations:** a Department of Cardiovascular Surgery, Chiayi Chang Gung Memorial Hospital, Puzi, Chiayi, Taiwan; b Department of Medicine, Chang Gung University, Taoyuan, Taiwan; c Department of Nursing, Chiayi Chang Gung Memorial Hospital, Puzi, Chiayi, Taiwan

**Keywords:** Modified FeneOS procedure, Hybrid pin penetration open stent technique, HIPPOS, Frozen elephant trunk procedure, Aortic arch

## Abstract

Total arch replacement using the frozen elephant trunk technique for acute type A aortic dissection involving the aortic arch remains technically demanding because of complex supra-aortic branch reconstruction and prolonged circulatory arrest. We developed a modified fenestrated open stent technique, termed the hybrid pin-penetration open stent (HIPPOS) technique, to simplify branch reconstruction while minimizing supra-aortic vessel manipulation. We retrospectively reviewed 15 consecutive patients who underwent total arch replacement using HIPPOS between May 2024 and May 2026 at a single institution by a single surgeon. Following deployment of a Frozenix open stent graft (Japan Lifeline), antegrade fenestrations were created under direct vision using needle penetration and sequential dilation, followed by reconstruction with balloon-expandable covered stent grafts. All procedures were successfully completed. Operative durations decreased with increasing numbers of fenestrations, reflecting procedural refinement and the surgical learning curve. Two perioperative deaths occurred in patients with profound preoperative shock, and one patient experienced a nondisabling stroke. No postoperative hoarseness, paraplegia, fenestration-related endoleak, or distal stent-induced new entry was observed. Follow-up computed tomography angiography confirmed satisfactory graft conformability and durable branch patency. HIPPOS is a reproducible modification of the frozen elephant trunk procedure with encouraging early clinical outcomes.

Type A aortic dissection involving the aortic arch is a life-threatening condition that presents significant technical challenges for cardiovascular surgeons. The frozen elephant trunk (FET) procedure is widely adopted as the preferred strategy for total arch replacement (TAR) in these patients. However, several limitations of conventional FET techniques remain. First, most commercially available endografts used in FET are not designed for direct surgical anastomosis, and suture-line bleeding may occur due to needle penetration through the graft material. In addition, mobilization of the left subclavian artery (LSCA) can increase the risk of recurrent laryngeal nerve injury.^1^ To address these issues, various hybrid techniques have been developed, including the fenestrated open stent technique (FeneOS)^2^ and branched stented anastomosis FET repair.^3^

Building on these approaches, we sought to further reduce LSCA manipulation and eliminate the need for direct suturing to the endograft. We therefore adopted open stent grafting in the FET procedure and introduced a modification of the FeneOS technique. In this report, we describe our modified approach as modified FeneOS procedure, or termed the hybrid pin-penetration open stent (HIPPOS) technique—and present early results regarding its safety and feasibility.

## Methods and surgical technique details

We reviewed 15 consecutive patients who underwent TAR for acute type A aortic dissection at our institution between May 2024 and May 2026. All procedures were performed by the same primary surgeon. Patients were stratified into three groups according to the number of fenestrations (zero, one, or two) created on the stent graft for supra-aortic vessel reconstruction. We collected the demographics data preoperatively, including age, gender, comorbidities, and conditions on arrival. Also, perioperative data including body circulation arrest time, cardiopulmonary bypass (CPB) time and cardiac arrest time were collected. Outcomes were measured with perioperative death, stroke, paraplegia, organ failure, hoarseness, and period of hospitalization.

All operations were carried out via median sternotomy under general anesthesia with the patient in the supine position. CPB was established through femoral arterial and right atrial cannulation. After cross-clamping the ascending aorta, antegrade histidine-tryptophan-ketoglutarate cardioplegia was administered through both coronary ostia. Systemic cooling was initiated, and lower-body circulatory arrest was instituted once the rectal temperature reached 28 °C. Antegrade selective cerebral perfusion was maintained via the innominate and left common carotid arteries using SP-GRIPFLOW circulation perfusion catheter (Fuji Systems Corporation).

The aortic arch was transected at zones 1, 2, or 3, depending on the preoperative fenestration strategy. A commercially available open stent graft (J Graft Frozenix, Japan Lifeline) was deployed under direct vision approximately 5 mm proximal to the transection site, with or without guidewire assistance. Sizing of the stent graft was based on the size of estimated distal landing zone on preoperative computed tomography angiography (CTA). Oversizing with discretion was suggested and was always between 0% and 10%. Fenestrations were created by puncturing the graft with an 18-gauge needle aiming the ostia of the target supra-aortic vessels, followed by guidewire passage (Terumo 0.035) and sequential dilation. During fenestration, the target vessel ostium was exposed without circumferential dissection or vascular control. Correct guidewire orientation was confirmed by direct visualization and tactile palpation of the guidewire and dilator within the target vessel before deployment of the branched stent graft. Balloon-expandable covered stents (BeGraft Peripheral, Bentley Innomed; or GORE VIABAHN VBX, W. L. Gore & Associates, Inc) were then advanced over the wire to reconstruct the corresponding branches (length 3.7 cm for BeGraft and 3.9 cm for VBX). The size of the branched fenestration stent graft was chosen according to preoperative CTA. Oversizing with 10% to 15% was suggested. Fenestrations were consistently placed within segments supported by the stent framework and never at the free, unstented free end of the graft (Fig 1). The branched stent grafts were deployed with approximately 5 mm of the proximal end protruding into the Frozenix graft to ensure adequate overlap. Following deployment, back bleeding from each reconstructed supra-aortic branch was assessed to confirm vessel patency. In the presence of brisk back bleeding and adequate cerebral oximetry, intraoperative angiography was not considered necessary. The Frozenix stent graft was secured to the native aortic wall with 15 to 20 interrupted 4-0 polypropylene sutures. A four-branched vascular graft (J Graft, Japan Lifeline) was subsequently anastomosed to the Frozenix device. Proximal anastomosis to the aortic root was completed, followed by direct anastomosis of any supra-aortic branches not reconstructed using fenestration stenting to the corresponding side branches of the J Graft.

**Fig 1:**
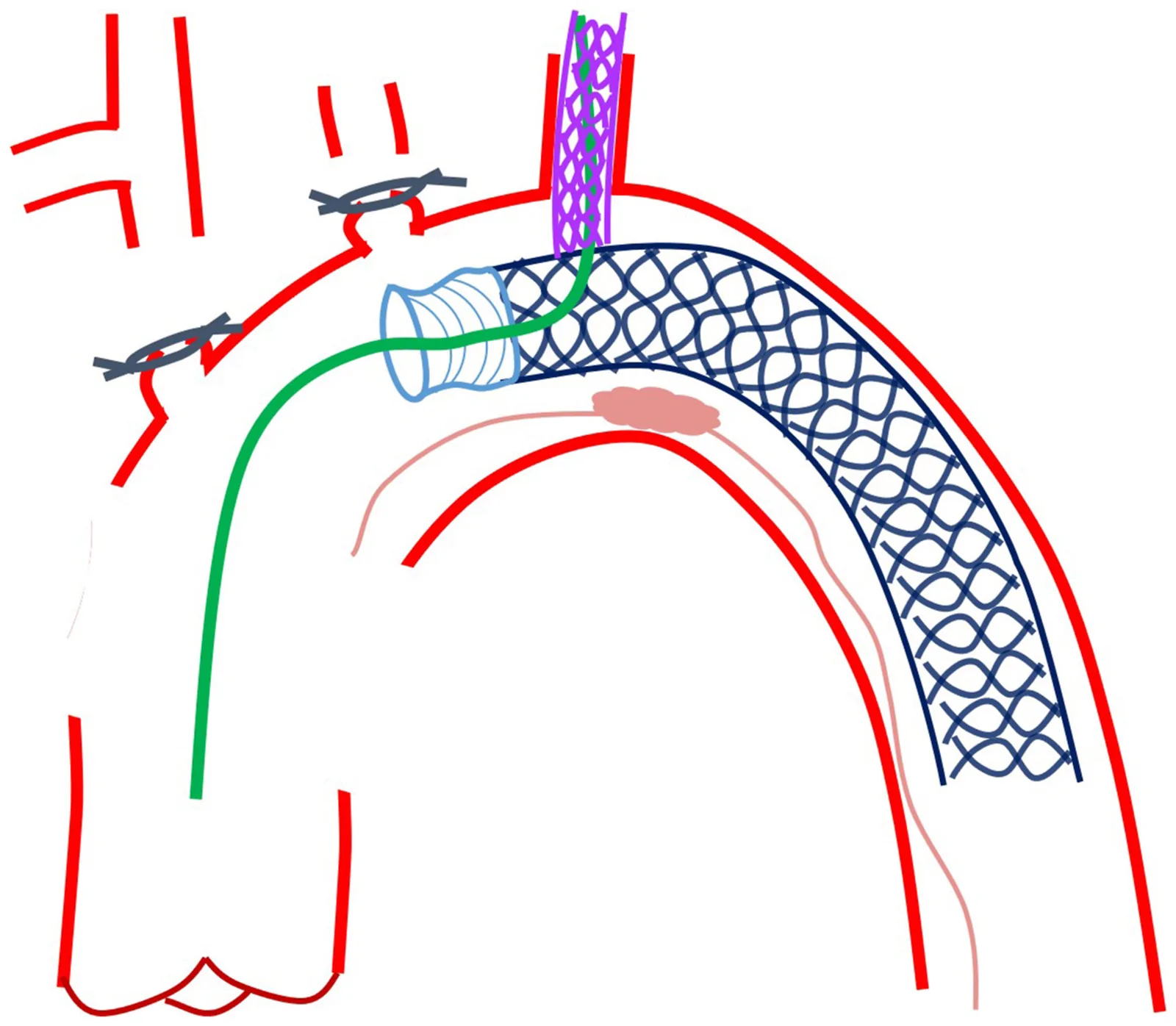
Diagram of key operation procedure of hybrid pin-penetration open stent (HIPPOS) technique (one-fenestration method). After Frozenix (*dark blue*) deployed in distal arch, a puncture hold was created at the inner side of the stent graft. With a Terumo 0.035 guidewire (*green*) passing through the puncture hole, a balloon-expandable stent graft (*purple*) was sent and deployed into target supra-aortic artery. The remaining supra-aortic arteries [innominate artery and left common carotid artery (LCCA)] were revascularized with open surgical bypass.

## Results

A total of 15 patients with acute type A aortic dissection underwent TAR using the HIPPOS technique. Patients were stratified according to the number of fenestrations created for supra-aortic branch reconstruction: no fenestration (n = 3), one fenestration (n = 7), and two fenestrations (n = 5). A Frozenix open stent graft and a four-branched J graft were used in all patients.

Preoperative patient condition and demographics were shown in Table I. Patients tended to be old with multiple comorbidities. Preoperative shock was common among patients. Perioperative data were presented in Table II. There was an obvious trend toward decreases in lower-body arrest duration, CPB time, and cardiac arrest duration with increasing numbers of fenestrations, especially between the group with two fenestrations and the other two groups. Postoperative CTAs were demonstrated in Fig 2. Kaplan-Meier analysis of overall survival is presented in Fig 3. Two patients with profound preoperative shock died within the early postoperative period, whereas the remaining patients continued regular clinical follow-up. Postoperative CTA was routinely performed 1 month after surgery and annually thereafter. Among the 13 surviving patients, postoperative CTA demonstrated no evidence of fenestration-related endoleak or distal stent-induced new entry (SINE) throughout the imaging follow-up period (Fig 3).

**Table I:** Demographics and preoperative condition of the patients in each group

Variables	0 fenestration (n = 3)	1 fenestration (n = 7)	2 fenestrations (n = 5)
Mean age, years	75.0	69.0	69.2
Sex, men/women	2/1	4/3	3/2
Preoperative condition			
Shock	1	2	1
Stroke	0	1	1
Diabetes mellitus	1	1	1
Hypertension	3	7	5
Renal failure	0	0	1
Ischemic heart disease	1	0	1

**Table II:** Perioperative data and outcomes of patients in each group

Variables	0 fenestration (n = 3)	1 fenestration (n = 7)	2 fenestrations (n = 5)
Average body circulation arrest duration, minutes	93.5 ± 14.7	84.6 ± 29.7	75.4 ± 17.6
Average CPB duration, minutes	267.0 ± 15.6	243.3 ± 42.6	196.6 ± 34.2
Average cardiac arrest duration, minutes	199.0 ± 11.3	180.6 ± 39.4	148.6 ± 25.7
Outcomes, n			
Perioperative death	1	1	0
Disabling stroke	0	0	0
Any stroke	0	0	1
Paraplegia	0	0	0
Renal failure	0	0	0
MV dependent	0	0	0
Hoarseness	0	0	0
Length of stay, day			
ICU	6.5 ± 2.1^a^	5.1 ± 2.3^a^	4.8 ± 2.1
Hospitalization	32.5 ± 3.3^a^	24.0 ± 9.8^a^	26.2 ± 10.8

*CPB*, Cardiopulmonary bypass; *ICU*, intensive care unit; *MV*, mechanical ventilation.

a ICU and hospital stay calculated from available survivors only.

**Fig 2:**
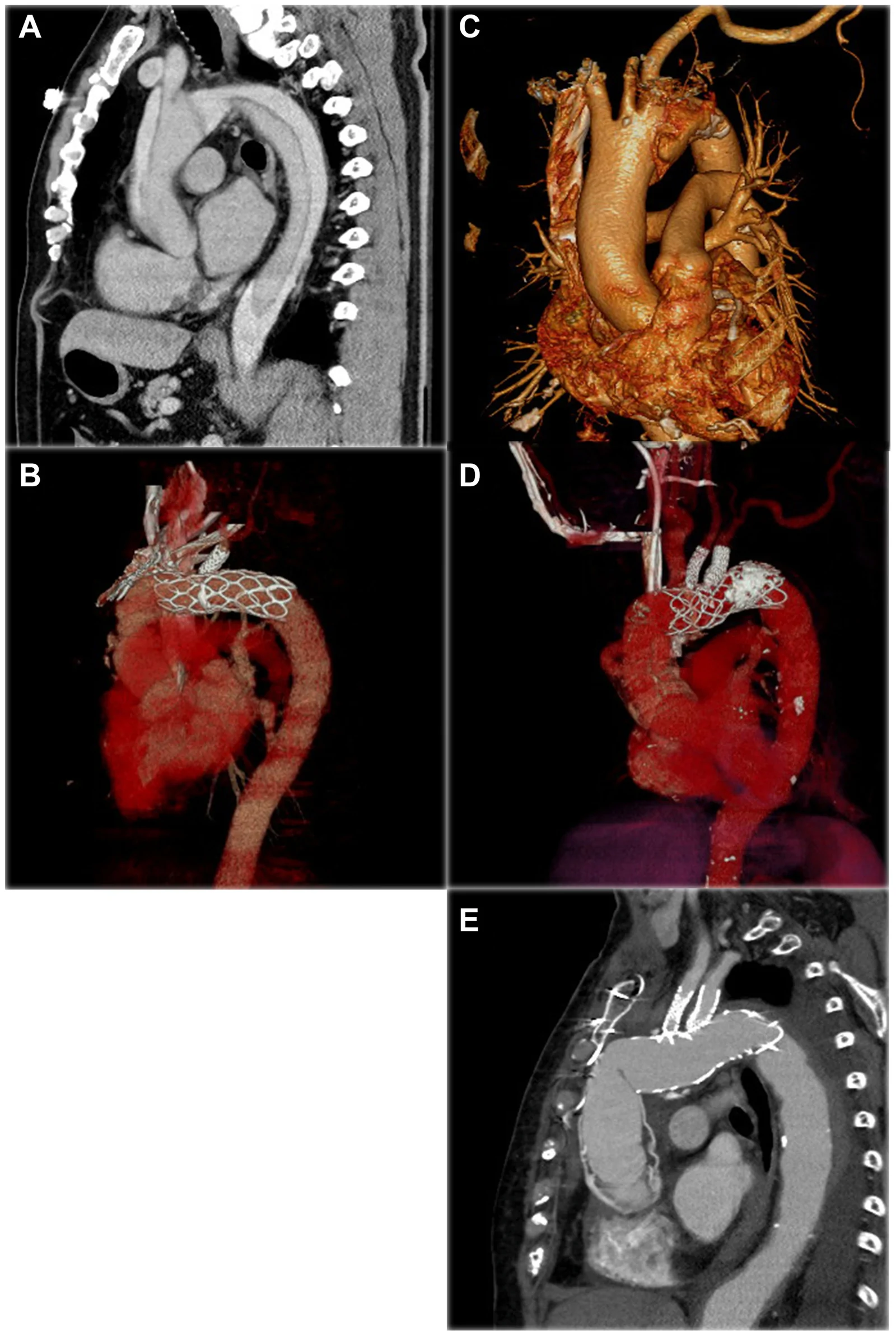
Comparison of preoperative and postoperative aorta computed tomography angiography (CTA) in hybrid pin-penetration open stent (HIPPOS) technique cases. **A** and **B,** Preoperative **(A)** and postoperative **(B)** CTA of a type A aortic dissection case. In panel A, the dissection flap extended from aortic arch proximally and distally. In panel B, after receiving HIPPOS technique with one fenestration, the Frozenix stent graft was conformed to the shape of the aortic arch and showed no sign of distal stent-induced new entry (SINE). The branched stent graft to left subclavian artery is patent. **C-E,** Preoperative **(C)** and postoperative **(D** and **E)** CTA of a type A aortic dissection patient. In panel A, the three-dimensional (3D) reconstruction image of aorta showed dissection flap involving the aortic arch and ascending aorta. After receiving HIPPOS procedure with two fenestrations, in both sagittal view **(E)** and 3D reconstruction **(D)** CTA, the Frozenix stent graft was conformed to the contour of aortic arch. Also, both stent grafts in supra-aortic arteries were patent. There was no endoleak presented at the site of stent graft fenestrations.

**Fig 3:**
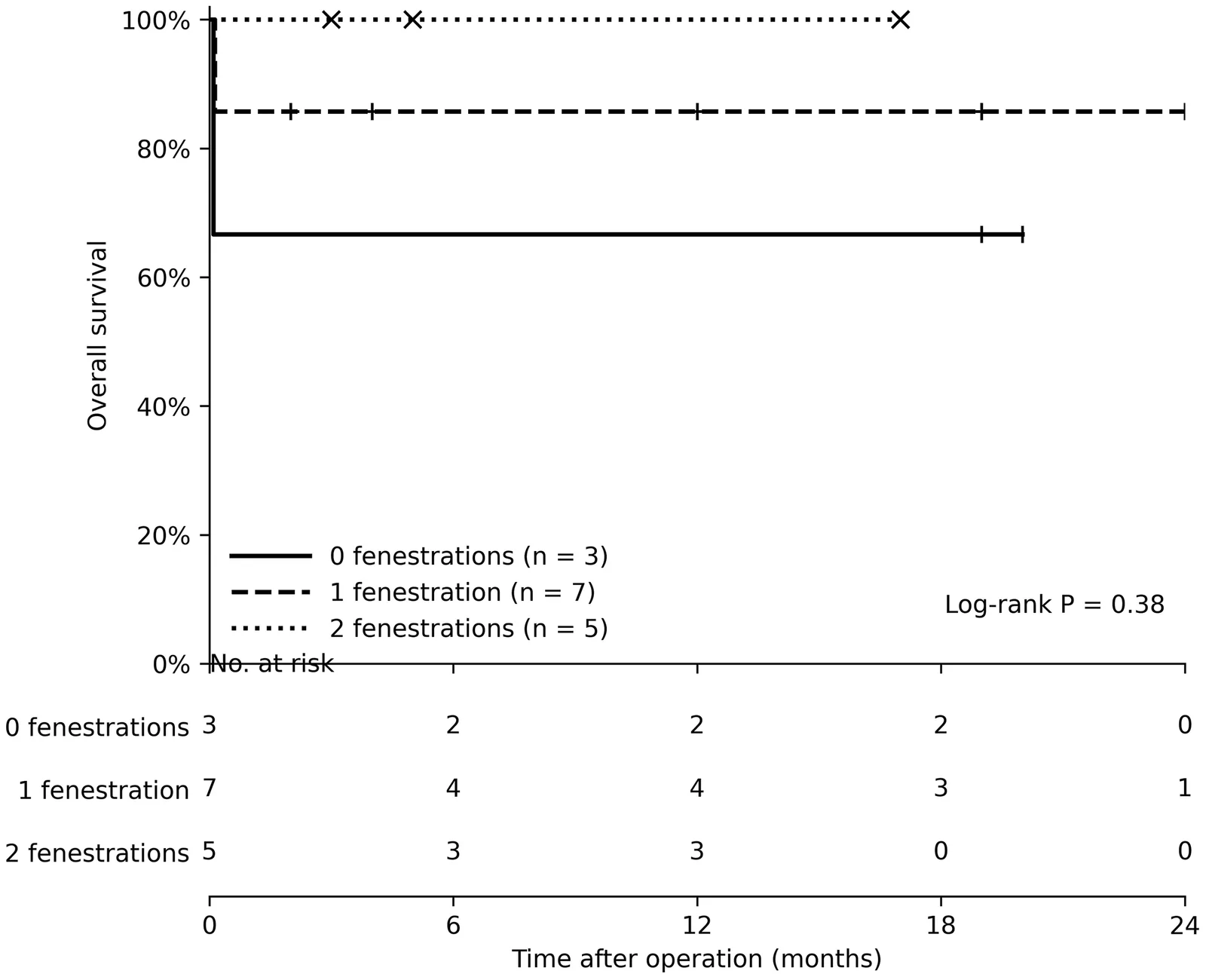
Kaplan-Meier estimates of overall survival according to the number of fenestrations. Vertical marks indicate censored observations. Numbers of patients at risk are shown below the graph.

## Discussion

The FET procedure is a technically demanding and time-intensive operation. Prolonged CPB and hypothermic circulatory arrest have been associated with an increased risk of postoperative neurological complications and respiratory failure.^4^^,^^5^ In addition, manipulation of the LSCA carries the potential for recurrent laryngeal nerve injury.^1^ Various modifications of the FET technique have been proposed to mitigate these risks.^2^^,^^3^ In developing our approach, we aimed to further optimize clinical outcomes by reducing operative complexity and minimizing the aforementioned complications. Before the introduction of the HIPPOS technique, hemiarch replacement was the standard surgical strategy at our institution because of its lower-perioperative morbidity and mortality.^6^ TAR was infrequently performed in the emergency setting. During the study period, no patients underwent conventional TAR other than the 15 consecutive patients treated with the HIPPOS technique. In the following section, we outline the technical distinctions between our modification and existing techniques, as well as discuss their potential advantages.

First, the HIPPOS technique further reduces the need for direct manipulation of the LSCA. In the original FeneOS procedure described by Okamura et al,^2^ fenestrations are created using a blade and scissors, and a manually constructed U-shaped vascular graft is applied externally to reinforce the fenestration site. In contrast, the HIPPOS approach secures the fenestration by deploying a balloon-expandable stent graft, thereby eliminating the need for external graft reinforcement. This enables a true “no-touch” strategy for supra-aortic vessels designated for revascularization through the open fenestration technique. By avoiding LSCA manipulation, the risk of recurrent laryngeal nerve injury may be significantly reduced. Consistent with this expectation, no cases of postoperative hoarseness were observed in our series (Table II).

We observed a progressive reduction in CPB, lower-body circulatory arrest, and cardiac arrest durations with increasing numbers of fenestrations. This trend likely reflects both a reduction in the number of surgical anastomoses required and the procedural refinement associated with the surgical learning curve as the HIPPOS technique evolved from zero to one and subsequently two fenestrations. The greatest reduction was observed after adoption of the two-fenestration strategy, suggesting that increasing familiarity with the technique and procedural streamlining contributed substantially to improved operative efficiency.

A potential concern regarding the use of open fenestration with branched stent grafts is the risk of impaired branch flow or endoleak at the fenestration sites. To address these concerns, we drew on the endovascular technique developed by Ohki et al,^7^ in which retrograde puncture from distal arterial access is used to fenestrate the aortic stent graft. Their method demonstrated favorable outcomes, including high patency rates and a low incidence of endoleak. In the HIPPOS technique, fenestration is performed antegrade under direct vision after aortic arch transection, allowing accurate deployment of branch stent grafts without fluoroscopy.

During the development phase of HIPPOS, we initially created a single fenestration for the LSCA in the first three cases. Postoperative assessment included bilateral upper-extremity blood pressure measurements and transcranial duplex ultrasound study to evaluate vertebral artery flow. All three patients demonstrated symmetric vertebral artery perfusion and equivalent blood pressure in both arms. Based on these encouraging findings, we subsequently extended the proximal landing zone of the Frozenix device to zone 1 and increased the number of fenestrations to two—targeting both the left common carotid artery (LCCA) and the LSCA. Follow-up CTA (Fig 2) confirmed patency of the fenestrations without evidence of endoleak. We had final number of HIPPOS procedures done with no fenestration (n = 3), one fenestration (n = 7), and two fenestrations (n = 5).

To standardize the HIPPOS procedure, we developed a practical algorithm for selecting the proximal landing zone of the aortic stent graft and determining the number of fenestrations required. As the primary objective of HIPPOS is to minimize manipulation of the LSCA, reconstruction of the LSCA by fenestration was considered the default strategy. Two-fenestration reconstruction was selected when both LCCA and LSCA could be reconstructed without excessive technical difficulty. Based on our experience, difficulty increased substantially when the distance between LCCA and LSCA ostia exceeded 2 cm, resulting in unfavorable branch stent orientation, prolonged deployment (>5 minutes), or the need to convert conventional surgical reconstruction. Therefore, when preoperative CTA demonstrated an interostial distance >2 cm, we preferred a single-fenestration strategy for the LSCA combined with conventional surgical anastomosis of the LCCA and innominate artery using the side branches of the four-branched J graft.

The presence of supra-aortic branch dissection alone was not considered a contraindication to the fenestration technique. Fenestration was abandoned only when the true lumen could not be reliably distinguished from the false lumen under direct vision. In anatomically challenging cases, placement of a guidewire from the left axillary or subclavian artery to the LSCA ostium before fenestration may facilitate accurate delivery and deployment of the branched stent graft. When fluoroscopic guidance is available, this adjunctive technique may further improve procedural accuracy.

During the development of the HIPPOS technique, we identified the Frozenix device (Fig 4) as the most suitable stent graft for this application. Frozenix is specifically designed for open surgical deployment and consists of a rounded nitinol stent structure covered by Dacron polyester fabric. Compared with conventional thoracic endovascular aortic repair endografts, the circular stent configuration of Frozenix provides greater space for creating fenestrations, reducing the likelihood that the branched stent graft will be compressed by the underlying frame. In addition, the Dacron fabric offers substantial resistance to shear forces during manual fenestration, thereby minimizing the risk of graft tearing adjacent to the fenestration site. A distinctive feature of the Frozenix design is the long Dacron “sleeve” attached proximally to the stent graft. Although not intended for use as a standard vascular prosthesis, this sleeve is versatile and can serve as reinforcement for the anastomosis between the Frozenix device and the four-branched J-Graft. In the HIPPOS technique, we routinely trim this sleeve to a length of approximately 1 cm and secure it to the native aortic wall using circumferential interrupted sutures.

**Fig 4:**
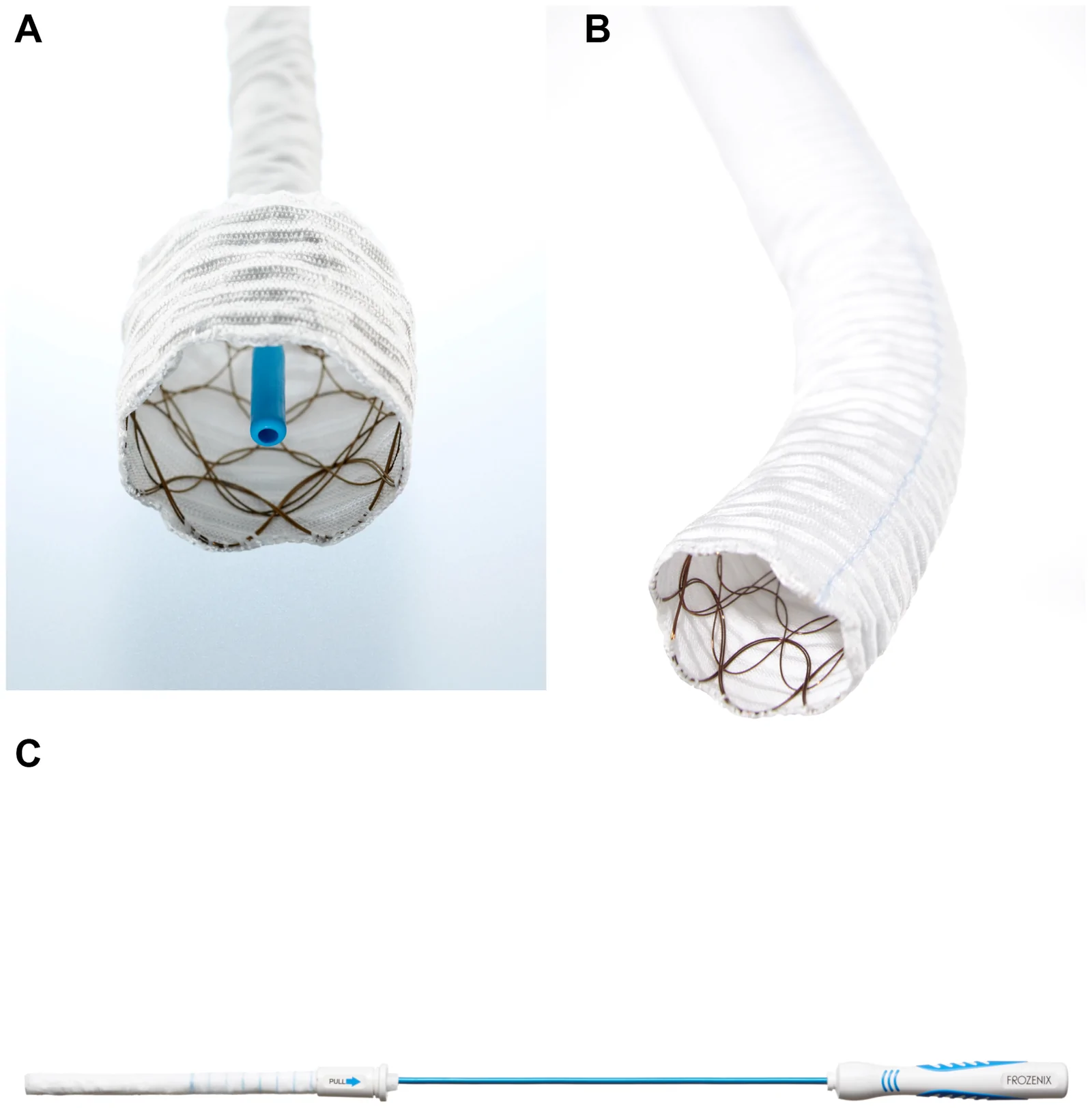
Photographs of the Frozenix open stent graft (J Graft Frozenix; Japan Lifeline). **A,** Distal end of the Frozenix device during partial deployment. The rounded nitinol stent framework enclosed within the Dacron graft is visible, illustrating the stent-supported segment where fenestrations are created in the hybrid pin-penetration open stent (HIPPOS) technique. **B,** Distal end of the Frozenix device after complete deployment. The fully expanded circular stent configuration provides adequate space for branch stent reconstruction while maintaining conformability to the aortic arch. **C,** Frozenix device mounted on its delivery system before deployment. The graft is deployed by pulling back the outer sheath, as indicated by the “PULL” marker. The blue centimeter markers along the delivery shaft facilitate positioning, and the thick blue marker identifies the proximal end of the stent-supported segment.

The FET procedure has been widely criticized for its potential risk of spinal cord ischemia. Although we did not routinely use preoperative spinal drainage in our patients, no cases of paraplegia occurred in our series. The Frozenix device is available in multiple lengths, ranging from 6 to 15 cm, allowing individualized selection based on preoperative CTA. In the HIPPOS technique, graft length is chosen with particular attention to minimizing distal coverage. We typically used a 9-cm Frozenix when the proximal landing zone was at zone 2, and a 12-cm graft when landing at zone 1. The inherent flexibility of the Frozenix design ensures excellent conformability to the curvature of the aortic arch and descending thoracic aorta, even without guidewire assistance. This characteristic enables the selection of the shortest feasible graft length, thereby reducing coverage of intercostal arteries and potentially lowering the risk of spinal cord ischemia. Follow-up CTA (Fig. 2) demonstrated that, even in patients with a long transverse arch, the Frozenix device appropriately conformed to the distal arch curvature without evidence of distal stent-induced new entry.

An alternative approach for selected patients with acute type A aortic dissection involving the aortic arch is total endovascular repair with in situ laser fenestration.^8^ Although this technique avoids open surgery, its application is generally limited to patients who are considered poor candidates for conventional surgical repair and who have favorable aortic root anatomy with an adequate proximal landing zone. The HIPPOS technique provides an alternative hybrid approach for patients who remain suitable candidates for open arch reconstruction.

### Limitation

The HIPPOS technique has been reported in a limited number of cases and was applied only in patients with type A aortic dissection.

## Conclusions

The HIPPOS technique appears to be a safe and reproducible approach for TAR. In this study, we applied the method to patients with type A aortic dissection involving the aortic arch and achieved favorable early outcomes, even in the most acute presentations. These encouraging results suggest that the technique may have broader applicability and could potentially be extended to other aortic arch pathologies in future practice.

## Ethics approval and consent to participate

All participants have agreed to be included in the study.

## Consent for publication

The authors have acquired informed consent for publication from the study subjects.

## Availability of data and materials

Data will be available from the corresponding author upon request.

## Declaration of generative AI and AI-assisted technologies in the writing process

During the preparation of this work, the authors used ChatGPT for language correction. After using this tool, the authors reviewed and edited the content as needed and take full responsibility for the content of the publication.

## Funding

None.

## Disclosures

None.
